# Methyl Jasmonate-Treated Pepper (*Capsicum annuum* L.) Depresses Performance and Alters Activities of Protective, Detoxification and Digestive Enzymes of Green Peach Aphid [*Myzus persicae* (Sulzer) (Hemiptera: Aphididae)]

**DOI:** 10.1093/jisesa/ieac074

**Published:** 2022-12-21

**Authors:** Xue Zhan, Ying Liu, Xiao Liang, Chunling Wu, Xiaoqiang Liu, Jun Shui, Yao Zhang, Ying Wang, Qing Chen

**Affiliations:** State Key Laboratory Breeding Base of Green Pesticide and Agricultural Bioengineering/Key Laboratory of Green Pesticide and Agricultural Bioengineering, Ministry of Education, Guizhou University, Guiyang 550025, China; Environment and Plant Protection Institute, Chinese Academy of Tropical Agricultural Sciences/Key Laboratory of Integrated Pest Management on Tropical Crops, Ministry of Agriculture and Rural Affairs, Haikou 571101, China; Sanya Research Academy, Chinese Academy of Tropical Agriculture Science/Hainan Key Laboratory for Biosafety Monitoring and Molecular Breeding in Off-Season Reproduction Regions, Sanya 572000, China; Environment and Plant Protection Institute, Chinese Academy of Tropical Agricultural Sciences/Key Laboratory of Integrated Pest Management on Tropical Crops, Ministry of Agriculture and Rural Affairs, Haikou 571101, China; Sanya Research Academy, Chinese Academy of Tropical Agriculture Science/Hainan Key Laboratory for Biosafety Monitoring and Molecular Breeding in Off-Season Reproduction Regions, Sanya 572000, China; Environment and Plant Protection Institute, Chinese Academy of Tropical Agricultural Sciences/Key Laboratory of Integrated Pest Management on Tropical Crops, Ministry of Agriculture and Rural Affairs, Haikou 571101, China; Sanya Research Academy, Chinese Academy of Tropical Agriculture Science/Hainan Key Laboratory for Biosafety Monitoring and Molecular Breeding in Off-Season Reproduction Regions, Sanya 572000, China; Environment and Plant Protection Institute, Chinese Academy of Tropical Agricultural Sciences/Key Laboratory of Integrated Pest Management on Tropical Crops, Ministry of Agriculture and Rural Affairs, Haikou 571101, China; Sanya Research Academy, Chinese Academy of Tropical Agriculture Science/Hainan Key Laboratory for Biosafety Monitoring and Molecular Breeding in Off-Season Reproduction Regions, Sanya 572000, China; Environment and Plant Protection Institute, Chinese Academy of Tropical Agricultural Sciences/Key Laboratory of Integrated Pest Management on Tropical Crops, Ministry of Agriculture and Rural Affairs, Haikou 571101, China; Sanya Research Academy, Chinese Academy of Tropical Agriculture Science/Hainan Key Laboratory for Biosafety Monitoring and Molecular Breeding in Off-Season Reproduction Regions, Sanya 572000, China; State Key Laboratory Breeding Base of Green Pesticide and Agricultural Bioengineering/Key Laboratory of Green Pesticide and Agricultural Bioengineering, Ministry of Education, Guizhou University, Guiyang 550025, China; Environment and Plant Protection Institute, Chinese Academy of Tropical Agricultural Sciences/Key Laboratory of Integrated Pest Management on Tropical Crops, Ministry of Agriculture and Rural Affairs, Haikou 571101, China; Sanya Research Academy, Chinese Academy of Tropical Agriculture Science/Hainan Key Laboratory for Biosafety Monitoring and Molecular Breeding in Off-Season Reproduction Regions, Sanya 572000, China; Environment and Plant Protection Institute, Chinese Academy of Tropical Agricultural Sciences/Key Laboratory of Integrated Pest Management on Tropical Crops, Ministry of Agriculture and Rural Affairs, Haikou 571101, China; Sanya Research Academy, Chinese Academy of Tropical Agriculture Science/Hainan Key Laboratory for Biosafety Monitoring and Molecular Breeding in Off-Season Reproduction Regions, Sanya 572000, China

**Keywords:** plant elicitor, induced resistance, aphid development, aphid reproduction, enzymatic alternation

## Abstract

Methyl jasmonate (MeJA) is a phytohormone that has been used to artificially induce plant resistance against multiple arthropod herbivores. However, it is still uncertain whether MeJA can trigger pepper plant resistance against *Myzus persicae* (Sulzer) (Hemiptera: Aphididae) (green peach aphid, GPA). In this study, we assessed the effects of different concentrations (0, 0.008, 0.04, 0.2, 1.0, and 5.0 mM) of MeJA-treated pepper on the development and reproduction performance of GPA to identify an appropriate concentration for vigorous resistance enhancement. MeJA dose was applied on the pepper to investigate the changes in activities of protective enzyme (superoxide dismutase, SOD; catalase, CAT; peroxidase, POD and polyphenol oxidase, PPO), detoxification enzymes (acetylcholinesterase, AchE; glutathione S-transferase, GSTs; cytocrome P450, CYP450, and carboxylesterase, CarE), and digestive enzymes (protease, PRO and amylase, AMY) in GPA. The results showed that all concentrations of MeJA-treated pepper significantly suppressed GPA performance, wherein 0.2 mM was the optimal concentration, as it presented the lowest intrinsic rate of increase (*r*_*m*_), finite rate of increase (*λ*), and the highest population doubling time (*Dt*) values. Furthermore, the protective enzymes (SOD and CAT), detoxification enzymes (GSTs, CYP450, and CarE), and AMY activities increased significantly in MeJA-treated groups than the control group, while the POD and PPO activities were remarkly inhibited under 0.2 mM treatment. These findings indicate that exogenous spraying of 0.2 mM of MeJA significantly enhanced pepper resistance against GPA. The result of this study suggests MeJA application can be used as a promising strategy in integrative management of this insect pest.

Pepper (*Capsicum annuum* L.) is one of the most important vegetables and spice crops due to its aroma, taste, flavor, and pungency (Eggink et al. 2012). In China, pepper is the largest and most valuable vegetable crop (Wang et al. 2018). A broad range of arthropod herbivores can cause damage to pepper production. *Myzus persicae* (Sulzer) (Hemiptera: Aphididae), known as the green peach aphid (GPA), is one of the most devasting species. By penetrating the plant tissue with its stylet, the GPA not only causes direct damage but also transmits several plant viruses, which cause even more considerable losses (Frantz et al. 2004, Züst and Agrawal 2016). To now, the control of GPA mainly relies on insecticides; however, the excessive use of insecticides may largely reduce the natural enemies population, besides leading to insecticide resistance (Tang et al. 2017, Chen et al. 2020). Hence, seeking effective and eco-friendly control strategies is quite necessary.

Jasmonic acid (JA) and its methyl ester (MeJA) and isoleucine conjugate (JA-Ile), referred to jasmonates (JAs), are important molecules in the regulation of many physiological processes such as plant growth, development, as well as plant responses to biotic and abiotic stresses (Dar et al. 2015, Ruan et al. 2019). Methyl jasmonate (MeJA) is the most well-studied plant elicitor. Since a previous study speculated that exogenous application of MeJA can induce plant resistance to insects (Farmer and Ryan 1990), researchers began to focus on the mechanism of enhancing plant resistance. A number of studies reveal that MeJA may elevate the activities of several defense enzymes (Boughton et al. 2006, Paudel et al. 2014, Jiang and Yan 2018) and cause the accumulation of toxic secondary metabolites (Rodriguez-Saona et al. 2001, Wei et al. 2021), as well as insect proteinase inhibitors in plants (Sripontan and Hwang 2016), or, on the other hand, releasing volatile organic compounds (VOCs) to attract natural enemies (James 2005, Shahabinejad et al. 2014, Yu et al. 2018). In addition, the impact of MeJA-pretreated plant on insect performance was also evaluated. For instance, MeJA-treated soybean [*Glycine max* (L.) Merr.] reduced pupal weight of soybean looper, *Chrysodeixis includens* (Walker) (Lepidoptera: Noctuidae), by 6.8% and delayed larval development by 14.3% (Chen et al. 2018). Another study showed that population of soybean aphids [*Aphis glycines* Matsumura (Hemiptera: Aphididae)] was reduced by 25 % in MeJA-pretreated soybean plants (Selig et al. 2016). Partial spray of MeJA on *Larix olgensis* seedlings strongly decreased the larval/pupal weights and survivals, as well as the fecundity of gypsy moth (*Lymantria dispar* (L.) (Lepidoptera: Erebidae)) (Jiang and Yan 2018). Additionally, tall fescue [(*Lolium arundinaceum* (Schreb.) S.J. Darbyshire)] exposed to MeJA significantly increased resistance to bird cherry-oat aphid [*Rhopalosiphum padi* (L.) (Homoptera: Aphididae)] by stimulating defense compounds produced by the plant (Simons et al. 2008). Although the visible deterioration on insect phenotype was investigated, knowledge about internal biochemical changes in insect was still quite limited.

In this study, pepper seedlings were pre-treated with different concentrations of MeJA, and the development and reproduction performance and life table parameters of GPA were evaluated during herbivory. Subsequently, further investigation on the activity changes of protective enzymes (SOD, CAT, POD, and PPO), detoxification enzymes (AchE, GSTs, CYP450, and CarE), and digestive enzymes (PRO and AMY) were conducted by applying the pepper with the MeJA concentration that presented the most robust inhibition on aphid performance. The study could preliminarily decipher the possible mechanism of MeJA-induced resistance in pepper from the feedback of insect perspective, and provide the eco-friendly management strategy for green peach aphid.

## Materials and Methods

### Aphid Rearing and Pepper Cultivation

The GPA was originally cultured and supplied by Shandong Academy of Agricultural Sciences. In our lab, it was reared on caged tobacco plants (Yunyan 87 variety) (24℃, 16: 8 hr light: dark photoperiod and 70% RH).

Pepper seeds (variety Dayangjiaojiao, Guangzhou Changhe Seed Co. LTD, Guangzhou, China) were soaked in 50% (V/V) commercial disinfectant for 5 min, then rinsed with distilled water five times, the seeds were placed in an incubator (with no light at 28℃, 70% RH). The germinated seeds were first planted in a 72-well seedling tray for 3 weeks, and then the seedlings were individually transplanted into small plastic pots (6 × 6 × 8 cm) with a 1:1 mixture of peat (Pindstrup Mosebrug A/S, Ryomgard, Denmar) and vermiculite. Plants were watered three times a week and maintained in growth chambers under the GPA rearing condition as described previously. Pepper seedlings with 7–8 fully developed leaves (approximately 20 cm height) were used to initiate the experiment.

### Methyl Jasmonate Treatment

MeJA (Sinopharm Chemical Reagent Co. Ltd., Beijing, China) was dissolved in ddH_2_O (containing 0.05% Tween-20 [V/V]) to prepare the stock solution (5.0 mM) following a published method (Bhavanam and Stout 2021). Furthermore, the stock solution was five-fold diluted with 0.05% Tween-20 water solution to get a series of treated solutions (0.008, 0.04, 0.2, and 1.0 mM, respectively), and the 0.05% Tween-20 water solution was used as control.

Pepper seedlings were sprayed with MeJA or control solutions using 100-mL pressurized hand sprayers until runoff; after spraying, seedlings were immediately covered with a sealed transparent plastic bag (70 × 100 × 70 cm). After 6 hr treatment, the sealed plastic bag was removed and to allow the wetted leaves to dry. The plants treated with different doses of MeJA were placed in separate growth chambers to minimize the effects of volatile MeJA.

### GPA Performance and Life Table Parameters

Five GPA female adults were carefully transferred to a MeJA pretreated pepper seedling with a fine paintbrush. After 12 hr, all the GPAs were removed but left only one newborn aphid. Inoculated leaf was covered with a clip leaf cage to prevent the nymph from escaping (Florencio-Ortiz et al. 2018). Thirty replicates (30 pepper seedlings) were performed for the treatments and control (five MeJA treated doses needed 150 seedlings, and 30 seedlings were used for control, respectively). All inoculated plants were cultured in the growth chamber under the GPA rearing condition. Individual aphid was checked daily for the survivorship. After the nymph became an adult, the GPA population was monitored and the newborn nymphs were recorded and removed daily (only one single adult on each tested seedling before it died). In addition, for each treatment or control, the total pre-ovipositional period (TPOP, from birth to producing the ﬁrst nymph), fecundity (the number of off-springs produced by each female), oviposition period, and longevity (from birth to death) were recorded on each plant until the last female adult died. In addition, the GPA population parameters were calculated according to the following formulations.

The net reproduction rate (*R*_*0*_) (the average number of offspring per female during its whole life cycle) was calculated as (Rasekh et al. 2021):


$$\begin{array}{l} l_{x}=\underset{j=1}{\overset{k}{\sum }}\;S_{xj} \end{array}$$

$$\begin{array}{l} m_{x}=\frac{\overset{k}{\underset{j=1}{\sum }}S_{xj}f_{xj}}{\overset{k}{\underset{j=1}{\sum }}S_{xj}} \end{array}$$

$$\begin{array}{l} R_{0}=\underset{x=0}{\overset{\infty }{\sum }}\;l_{x}m_{x} \end{array}$$

where *k* denotes the number of stages, *x* = age in days, *j* = stage, *l*_*x*_ = age-specific survival rate, *m*_*x*_ = age-specific fecundity, *S*_*xj*_ = age-stage-specific survival rates, *fxj* = age-stage-specific fecundity.

The intrinsic rate of increase (*r*_*m*_) (the rate of population increase per unit time), finite rate of increase (*λ*) (the number of offspring added to the population per female per unit time), mean generation time (*T*) (the time elapsed between the birth of the parents and the birth of the offspring) and the population doubling time (*D*_*t*_) (the time necessary for the population to double) were calculated as:


$$\begin{array}{l} \underset{x=0}{\overset{\infty }{\sum }}\;e^{-r_{m}(x+1)}l_{x}m_{x}=1 \end{array}$$

$$\begin{array}{l} T=\frac{\ln\left(R_{0}\right)}{r_{m}} \end{array}$$

$$\begin{array}{l} \lambda =e^{r_{m}} \end{array}$$

$$\begin{array}{l} D_{t}=\frac{\ln\left(2\right)}{r_{m}} \end{array}$$

### Enzymatic Activity Assay

Pepper seedlings were treated using 0.2 mM of MeJA solutions and control solution as described above, and then 30 GPA adults were inoculated onto each pre-treated plant and caged. For enzyme analyses, the 30 aphids were sampled on 1, 2, 4, 8, 10, and 12 d after treatment, and placed in 1.5 ml centrifuge tube and stored at −80℃ before use. Each set of 30 aphids on each seedling was used as a replicate and each treatment was replicated three times.

Activities of protective enzyme (SOD, CAT, POD, and PPO), detoxification enzymes (AchE, GSTs, CYP450, and CarE), and digestive enzyme (PRO and AMY) were determined in soluble proteins extracted from the treated adult aphids (3 replicates/treatment/d). Thirty adult aphids were homogenated with a miniature handheld homogenizer in 1 ml ice-cold 1× phosphate buffered solution (pH 7.2–7.4). The homogenate was then centrifuged at 12,000 × *g* for 15 min at 4°C. The supernatant was directly used as a crude extract for activity assay.

The activities of the 10 tested enzymes were all measured according to the insect enzyme immunoassay kits (ml036253 [SOD], ml062687 [CAT], ml062688 [POD], ml062744 [PPO], ml022715 [AchE], ml076338 [GSTs], ml062675 [CYP450], ml036265 [CarE], ml062741 [protease], and ml062684 [amylase]), respectively, Shanghai Enzyme-linked Biotechnology Co. Ltd, Shanghai, China). The detection principle and operation method of each kit were similar, in which the Sandwich ELISA (monoclonal antibody of insect enzyme-tested aphid enzyme-horseradish peroxidase (HRP) labeled insect enzyme complex) was used. A 50 μl aliquot (10 μl of tested enzyme solution + 40 μl of supplied sample diluent) was transferred to the antibody-coated 96-well plate and the enzymatic activity was performed as described by the manufacturer’s protocol: the plate was first incubated at 37℃ for 30 min, then the plate was washed by the washing solution for five times; after that, 50 μl of HRP-labeled antibody was added to the sample well, and then we repeated the incubation and washing step. After washing, 100 μl of TMB (Tetramethylbenzidine) chromogen solution was added and incubated for another 10 min at 37℃. Finally, 100 μl of stop solution was added and incubated at 37℃ for 15 min, and the optical density (OD) value was measured using the Tecan Multimode Reader Platform (Spark M200, Grading, Austria) with a wavelength of 450 nm. The linear standard curve was established (using the standard solution within the kit, the series of standard activities of the standard solutions (U/L) were set as ’X’, and the corresponding OD values were set as ‘Y’) in parallel with the tested samples. The activity of each enzyme was presented as U/L (calculated via the standard curve).

### Statistical Analysis

The data were analyzed using GraphPad Prism software 8.0 (GraphPad Software, San Diego, CA) and Statistical software SPSS 19.0 (SPSS Inc., Chicago, IL) after checking for normality and homogeneity of variance. When necessary, data in our study were transformed to normalize the error variances using log or square-root transformation. When the pepper plants were treated with different concentrations of MeJA and control solution, the effects of MeJA treatments on fecundity, total pre-ovipositional period, oviposition period, longevity, and life table parameters of GPA were analyzed using one-way ANOVA, followed by Tukey’s test. For the GPA survival analysis, the log-rank (Mantel–Cox) test was used to analyze the significance between different MeJA treatments and control. When the pepper plants were treated with 0.2 mM of MeJA and control solution, a generalized linear mixed model (GLMM) was used to analyze the effects of MeJA application, treated time, and interactions between these two variables on various enzymatic activities of GPA, GLMM was implemented by SPSS GENLINMIXED, with a robust estimation method for standard errors (Huber-White sandwich estimator) to account for heterogeneity of variances. Moreover, a one‑sample *t*‑test was used to analyze enzymatic activity between MeJA treatment and control. Finally, Pearson’s correlation was applied between reproduction indexes (Total pre-ovipositional period, fecundity, oviposition period) and activities of 10 enzymes.

## Results

### Aphid Performance on MeJA-Treated Seedlings

The fecundity (*F*_5, 174_ = 61.9, *P* < 0.001) and oviposition period (*F*_5, 174_ = 18.1, *P* < 0.001) of GPA were all significantly decreased in the MeJA-treated seedlings compared with those in control seedlings (Table 1), and the effect of 0.2 mM, 1.0 mM and 5.0 mM MeJA treatment on aphid fecundity was significantly greater than that of the 0.008 mM and 0.04 mM MeJA treatments, but no difference was observed among these three concentrations. Furthermore, TPOP was significantly prolonged when fed with MeJA-treated pepper seedlings as compared to control (*F*_5, 174_ = 15.2, *P* < 0.001). In addition, the effect of 0.2 mM MeJA treatment on TPOP was significantly greater than that of the 0.008 mM and 0.04 mM MeJA treatments. Longevity was not significantly affected (*F*_5, 174_ = 0.6, *P* = 0.73).

**Table 1.** The mean (±SE) fecundity, total pre-ovipositional period, oviposition period, and longevity of green peach aphid fed on pepper treated with different concentrations of MeJA

Concentration(mM)	Fecundity	Total pre-ovipositional period (d)	Oviposition period (d)	Longevity (d)
0.0	50.4 ± 1.7 a	6.3 ± 0.1 d	16.0 ± 0.3 a	24.3 ± 0.4 a
0.008	31.2 ± 1.1 b	6.9 ± 0.1 bc	14.0 ± 0.2 bc	23.4 ± 0.6 a
0.04	32.0 ± 1.2 b	6.7 ± 0.1 c	14.3 ± 0.3 b	24.0 ± 0.6 a
0.2	26.2 ± 1.2 c	7.3 ± 0.1 a	13.1 ± 0.4 c	23.5 ± 0.6 a
1.0	25.6 ± 1.1 c	7.2 ± 0.1 ab	13.1 ± 0.4 c	24.2 ± 0.6 a
5.0	25.0 ± 0.9 c	7.2 ± 0.1 ab	13.1 ± 0.4 c	23.4 ± 0.6 a

Means (SE) within a column followed by the same letter are not significantly different according to Tukey’s HSD test at *P* < 0.05.

### Life Table Parameters Analysis

**Fig. 1.**
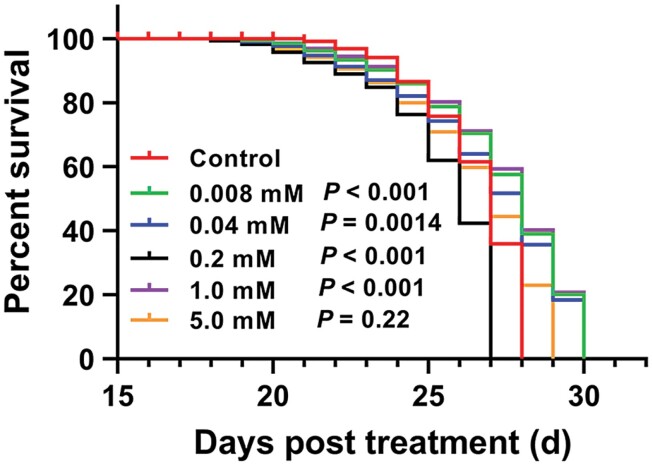
Survivorship curves of GPA (*n* = 30) fed on pepper treated with different concentrations of MeJA. The difference in survivorship curves was analyzed using the log-rank (Mantel–Cox) test and the data of control group was used as the reference.

When GPA fed on MeJA pretreated pepper, the survivorship curves showed similar trends; in particular, only the 0.2 mM of MeJA treatment presented a shorter curve compared with control and other treated concentrations (Fig. 1), indicating inhibition effect on the survival of GPA under the concentration of 0.2 mM. In addition, the intrinsic rate of increase (*r*_*m*_), finite rate of increase (*λ*), and net reproduction rate (*R*_*0*_) were all significantly reduced under MeJA treatment compared with control, and similarly, the minimum values of *r*_*m*_, *λ* were also obtained at 0.2 mM of MeJA treatment (0.2466 and 1.2798, respectively). Moreover, all the MeJA treatments significantly increased the population doubling time (*Dt*) and the *Dt* yielded the highest value (2.8160) at 0.2 mM of MeJA treatment (Table 2). In summary, as 0.2 mM treatment presented the most promising potential in impeding the GPA development and reproduction, this concentration was considered as the optimal concentration for further experiments.

**Table 2.** Life table parameters (mean ± SE) of green peach aphid fed on pepper treated with different concentrations of MeJA

Concentration (mM)	Intrinsic rate of increase (*r*_*m*_) (d^−1^)	Finite rate of increase (*λ*) (d^−1^)	Net reproduction rate (*R*_0_) (offspring)	Mean generation time (*T*) (d)	Population doubling time (*Dt*) (d)
0.0	0.2951 ± 0.0025 a	1.3433 ± 0.0034 a	50.5 ± 1.7 a	13.3 ± 0.2 a	2.35 ± 0.02 c
0.008	0.2690 ± 0.0028 b	1.3088 ± 0.0037 b	31.6 ± 1.1 b	12.8 ± 0.2 a	2.58 ± 0.03 b
0.04	0.2696 ± 0.0029 b	1.3096 ± 0.0038 b	32.2 ± 1.2 b	12.9 ± 0.2 a	2.58 ± 0.03 b
0.2	0.2466 ± 0.0020 c	1.2798 ± 0.0026 c	26.3 ± 1.2 c	13.2 ± 0.3 a	2.82 ± 0.02 a
1.0	0.2542 ± 0.0022 c	1.2895 ± 0.0029 c	25.6 ± 1.1 c	12.7 ± 0.2 a	2.73 ± 0.02 a
5.0	0.2517 ± 0.0022 c	1.2864 ± 0.0028 c	25.0 ± 0.9 c	12.8 ± 0.2 a	2.76 ± 0.02 a

Means (SE) within a column followed by the same letter are not significantly different according to Tukey’s HSD test at *P* < 0.05.

### Activities of Protective Enzymes in GPA Feeding on Seedlings Treated With MeJA

**Fig. 2.**
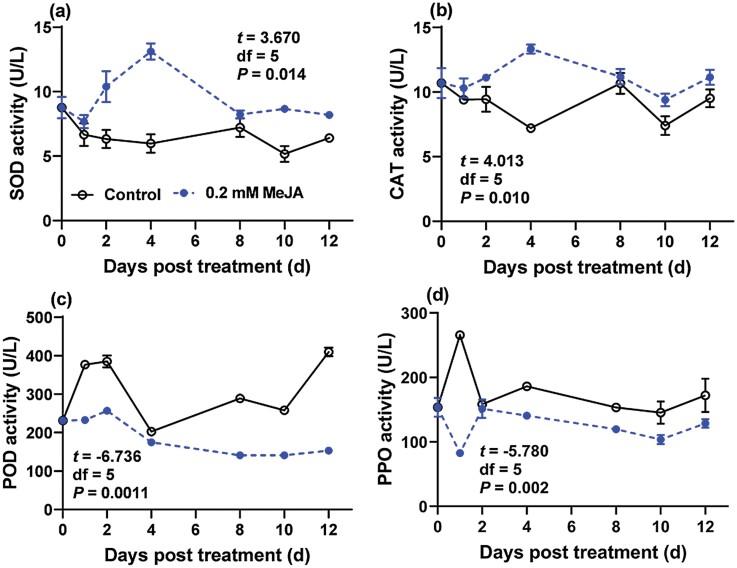
Effects of MeJA-treated pepper seedlings on the protective enzyme activities of GPA at 1, 2, 4, 8, 10, and 12 d after treatment. One‑sample *t*‑test was used to analyze the significance of enzymatic activity between MeJA treatment and control, besides, the *t,* df*, P* values were embedded in the each panel.

The GLMM analysis on activities of protective enzymes indicated a significant effect of MeJA treatment on SOD, CAT, POD, and PPO activities of GPAs. In addition, there was no significant effect of time as well as MeJA treatment-time interaction on protective enzyme activities (except PPO) (Table 3). Furthermore, one‑sample *t*‑test showed the activities of SOD and CAT were significantly higher than the mean value of those in control GPA, by contrast, the activities of POD and PPO were significantly inhibited (Fig. 2).

**Table 3.** Generalized linear mixed models (GLMMs) evaluating the effect of MeJA, time, and their interactions on green peach aphid enzymatic activities

Variables		Factors	Estimate	SE	95% CI	*P*
Protective enzyme	SOD activity	MeJA	3.89	0.92	2.00, 5.77	**<0.001**
		Time	−0.039	0.089	−0.22, 0.14	0.67
		MeJA*Time	−0.12	0.13	−0.38, 0.14	0.35
	CAT activity	MeJA	2.63	0.75	1.09, 4.16	**0.0015**
		Time	−0.017	0.074	−0.17, 0.13	0.81
		MeJA*Time	−0.069	0.10	−0.28, 0.14	0.51
	POD activity	MeJA	−83.56	34.55	−153.74, −12.98	**0.022**
		Time	−1.08	3.32	−7.83, 5.68	0.75
		MeJA*Time	−8.54	4.69	−18.09, 1.01	0.078
	PPO activity	MeJA	−75.99	11.93	−100.30, −51.69	**<0.001**
		Time	−5.27	1.18	−7.68, −2.86	**<0.001**
		MeJA*Time	4.07	1.67	0.66, 7.48	**0.021**
Detoxification enzyme	AchE activity	MeJA	9.97	13.09	−16.70, 36.64	0.45
		Time	1.81	1.24	−0.72, 4.35	0.16
		MeJA*Time	2.12	1.76	−1.47, 5.70	0.24
	GSTs activity	MeJA	388.41	32.64	321.92, 454.91	**<0.001**
		Time	5.41	3.20	−1.12, 11.93	0.10
		MeJA*Time	−18.82	4.53	−28.05, −9.60	**<0.001**
	CYP450 activity	MeJA	164.16	13.86	135.92, 192.40	**<0.001**
		Time	0.052	1.70	−3.41, 3.51	0.98
		MeJA*Time	−4.55	2.40	−9.44, 0.34	0.067
	CarE activity	MeJA	18.77	4.21	10.20, 27.34	**<0.001**
		Time	−3.99	0.42	−4.83, −3.14	**<0.001**
		MeJA*Time	1.29	0.59	0.092, 2.48	**0.036**
Digestive enzyme	AMY activity	MeJA	335.69	77.29	178.27, 493.11	**<0.001**
		Time	8.13	7.64	−7.43, 23.69	0.30
		MeJA*Time	20.66	10.80	−1.35, 42.66	0.065
	PRO activity	MeJA	10.26	5.34	−0.63, 21.15	0.064
		Time	0.18	0.53	−0.89, 1.24	0.74
		MeJA*Time	0.089	0.74	−1.42, 1.60	0.91

### Activities of Detoxification Enzymes in GPA Feeding on Seedlings Treated With MeJA

**Fig. 3.**
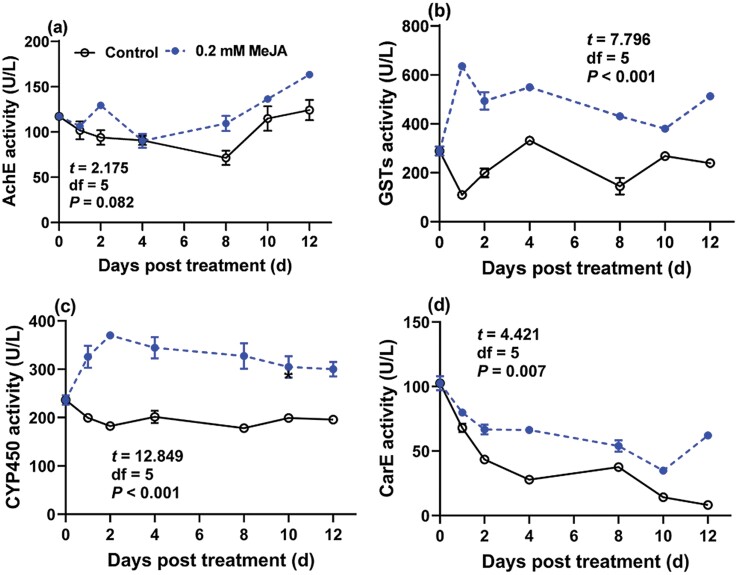
Effects of MeJA-treated pepper seedlings on the detoxification enzyme activities of GPA at 1, 2, 4, 8, 10, and 12 d after treatment. One‑sample *t*‑test was used to analyze the significance of enzymatic activity between MeJA treatment and control, besides, the *t,* df*, P* values were embedded in the each panel.

The GLMM analysis on activities of detoxification enzymes indicated a significant effect of MeJA treatment on GSTs, CYP450, and CarE activities of GPAs. In addition, there was a significant effect of time on CarE activity and significant MeJA treatment-time interaction on GSTs and CarE activities (Table 3). Furthermore, one‑sample *t*‑test showed GSTs, GSTs, and CarE activities of GPAs fed on MeJA-treated pepper were significantly higher than the mean value of those in control GPA (Fig. 3).

### Activities of Digestive Enzymes in GPA Feeding on Seedlings Treated With MeJA

**Fig. 4.**
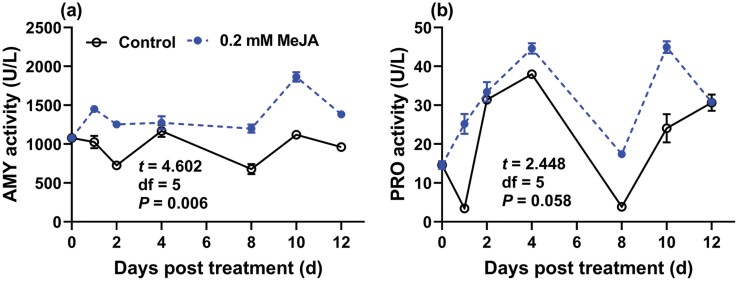
Effects of MeJA-treated pepper seedlings on the digestive enzyme activities of GPA at 1, 2, 4, 8, 10, and 12 d after treatment. One‑sample *t*‑test was used to analyze the significance of enzymatic activity between MeJA treatment and control, besides, the *t,* df*, P* values were embedded in the each panel.

The GLMM analysis on activities of digestive enzymes indicated a significant effect of MeJA treatment on AMY activity of GPA and there was neither significant effect of time nor MeJA treatment-time interaction on AMY and PRO activities (Table 3). Furthermore, one‑sample *t*‑test showed AMY activity of GPA fed on MeJA-treated pepper was significantly higher than the mean value of those in control GPA (Fig. 4).

### Pearson’s Correlation Analysis of Pairwise Comparisons of All Variables

**Fig. 5.**
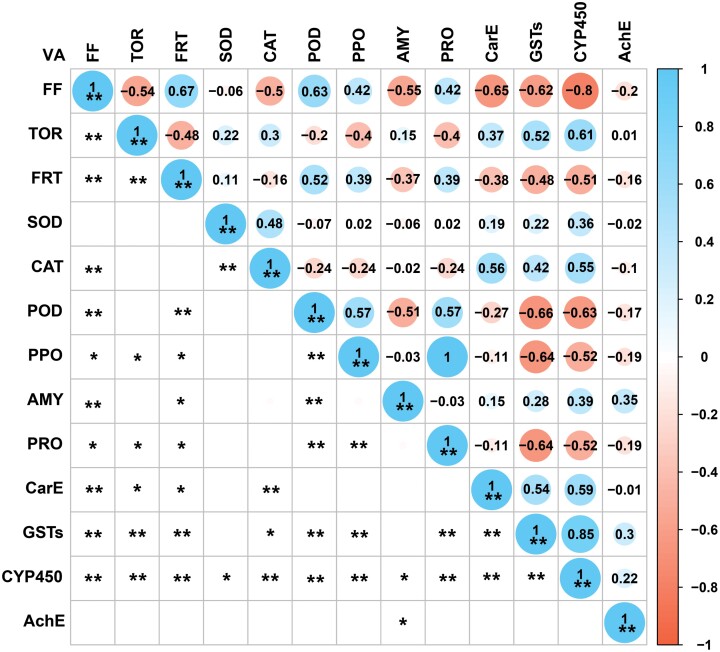
Pearson correlation coefficients between reproduction indexes (total pre-ovipositional period, fecundity, oviposition period) and activities of ten enzymes. VA, variable; FF, female fecundity; TPOP, total pre-ovipositional period; OP, oviposition period. * represents significance at *P* < 0.05; **, *P* < 0.01.

Fecundity correlated highly positively with the activities of POD (*r* = 0.63, *P* < 0.001), and correlated highly negatively with the activities of CYP450 (*r* = −0.80, *P* < 0.001), CarE (*r* = −0.65, *P* < 0.001), GST_S_ (*r* = −0.62, *P* < 0.001) and AMY (*r* = −0.55, *P* < 0.001). TPOP correlated highly positively with the activities of CYP450 (*r* = 0.61, *P* < 0.001) and GST_S_ (*r* = 0.52, *P* = 0.0012). The oviposition period correlated highly positively with POD activity (*r* = 0.52, *P* = 0.0013) and highly negatively with CYP450 activity (*r* = −0.51, *P* = 0.0015) (Fig. 5).

## Discussion

Spraying MeJA can induce plant resistance to aphids, which was demonstrated on several cultivated crops including wheat (Slesak et al. 2001), potato (Brunissen et al. 2010), tall fescue (Simons et al. 2008) and soybean (Selig et al. 2016). However, whether exogenous MeJA may strengthen pepper defense response against GPA was not clear. In the present study, we found that GPA feeding on MeJA-treated pepper seedlings would lead to significant decrease of the mean female fecundity, oviposition period and prolonged the total pre-ovipositional period. Our results corroborated that exogenous application of MeJA at multiple concentrations can sufficiently inhibit GPA reproduction. These findings agree with a previous study in which reduced rates of GPA population growth on MeJA-treated (7.5 mM) tomato plants (*Lycopersicon esculentum* cv Trust) were found(Boughton et al. 2006). By contrast, another study stated that MeJA-treated (5.0 mM) wheat deterred the grain aphid [*Sitobion avenae* (F.) (Hemiptera: Aphididae)] colonization processes and feeding behavior, but had no significant effects on aphid development, daily fecundity, intrinsic growth rate and population growth (Cao et al. 2014). The above phenomenon indicated that the exogenous MeJA-driven plant defense exhibited an insect-and plant-dependent pattern (Yang et al. 2022).

Different MeJA concentration possessed distinct impact in triggering plant resistance. In general, the concentration must be beyond the ‘threshold’, allowing MeJA to work sufficiently. However, very high concentrations would present a negative effect on plant and attenuate the resistance performance, as several studies have shown that excessive spraying of MeJA will cause potential phytotoxicity to plants e.g., negative impacts on plant growth and flowering, reducing yield (Boughton et al. 2006, Kraus and Stout 2019, Bhavanam and Stout 2021). In studies aimed to explore the appropriate applied MeJA, usually, the intermediate concentrations rather than the low or the high ones showed the best effect. The influence of three exogenous MeJA doses under field conditions was investigated on the abundance of the cotton pest predators, and results showed that the intermediate MeJA concentration had an attractive effect on the predators *Chrysoperla carnea* (Stephens) (Neuroptera: Chrysopidae) (Tonğa et al. 2022). In the present study, we considered 0.2 mM as the most suitable concentration of MeJA based on the following reasons. First, the dose used must show a significant effect on impeding the reproduction performance of GPA, in this study, all the treated concentrations presented this inhibition effect, and particularly, the concentrations of 0.2, 1.0, and 5.0 mM (higher concentration group) showed better inhibition than that of 0.008 mM and 0.04 mM (lower concentration group). Moreover, when conducting survival and life table parameters analysis, we found that the 0.2 mM of MeJA treatment presented the shortest survivorship curve, the lowest *r*_*m*_ and *λ*, and the highest *Dt*, and these indexes were recognized as important indicators evaluating the level of plant resistance to insects. Given the promising effect in enhancing pepper resistance, inhibiting GPA growth, and to avoid the potential negative influence on pepper growth, 0.2 mM of MeJA was finally selected for GPA enzymatic assay.

MeJA, as an important plant elicitor, can reprogram nutrient components and defense-related compounds to alter tissue palatability, which hinders herbivore digestion, development, and reproduction (Tan et al. 2011, Sripontan and Hwang 2016, Wei et al. 2021, Yan et al. 2021, Yang et al. 2022). To meet their nutritional requirements, insects can usually adjust their feeding habits (Cao et al. 2014), digestive physiology (War and Sharma 2014), host selection (Slesak et al. 2001, Sanches et al. 2017), and oviposition behavior (Disi et al. 2017). Therefore, we further characterized the enzyme activity changes of GPA feeding on MeJA-treated pepper seedlings, and further elucidated the enzyme-based plant resistance mechanism from the insect perspective.

SOD, CAT and POD are important antioxidative enzymes in insects. SOD converts toxic superoxide radicals into hydrogen peroxide and oxygen. CAT and POD are activated to catalyze hydrogen peroxide into water and oxygen (Felton and Summers 1995, Natalello et al. 2007, Lushchak 2014). The three enzymes can assist each other in scavenging reactive oxygen species and play a defensive role against free radicals. In the present study, alterations of SOD, CAT, and POD activity of GPA suggested that feeding on MeJA-treated pepper seedlings induced oxidative stress. More specifically, the activities of SOD and CAT were significantly activated, while the activity of POD was significantly inhibited, these results were partially consistent with a previous study, in which larvae of *Clostera anachoreta* (F.) (Lepidoptera: Notodontidae) fed with MeJA-treated (0.01 mM) poplar trees (*Populus* × *euramericana* variety ‘Nanlin895’) (Gu et al. 2018), SOD, CAT, and POD were all induced. Presumably, the antioxidant system was overwhelmed by severe oxidative damage of reactive oxygen species (ROS) (Mwaanga et al. 2014, Liu et al. 2020), which were probably triggered by the elevated toxic chemicals via the JA pathway, thus, resulting in the decrease of certain protective enzymes, i.e., POD. Nevertheless, further investigation is needed to elucidate the relationship between the changes of metabolites involved in defense and the alterations in the activity of antioxidant enzymes in GPA.

PPO plays an important role in insect metamorphosis development and immune defense. PPO is not only involved in melanin formation and sclerotization of the insect cuticle and wound healing, but also plays a role in the reaction as a nonself-recognition system in defense against parasites (Xiao et al. 2008). In this study, the PPO activity of GPA significantly decreased in MeJA-treated groups compared with the control group. This result agreed with the decrease of PPO activity in the cotton bollworm [*Helicoverpa armigera* (Hünber) (Lepidoptera: Noctuidae)] when fed on JA-treated (0.01, 0.1, and 1.0 mM) cotton plant leaves (Yang et al. 2022). This result may be unfavorable to GPA, as a previous study stated that the pathogen recognition, defense signal modulation, and transduction led to the expression of various effectors, which depended on PPO melanization (Li et al. 2017).

During the coevolution of insects and plants, insects have developed a set of mechanisms to resist plant defense responses (Erb and Reymond 2019). When insects feed on host plants, several toxic secondary substances were absorbed, and they would cope with the foreign chemicals by changing the activities of detoxification enzymes (Yang et al. 2022), such as mixed function oxidases (MFO), AchE, GSTs, and CarE (Prapanthadara et al. 2000). CYP450 is an important component of MFO and can be induced during toxicity stress. Insect adaptability on host plant can be improved by increasing the activity of detoxification enzymes. In the present study, the activities of GSTs, CYP450, and CarE in GPA reared on MeJA-treated pepper seedlings were significantly activated. The activities of CarE and GSTs in *Monolepta hieroglyphica* (Motschulsky) (Coleoptera: Chrysomelidae) were significantly inhibited when the insects fed on MeJA-treated (1.0 mM) rose *Rosa rugosa* Thunb. ‘Plena’ leaves (Yan et al. 2021). JAs treatment might activate the JA signaling pathway and induce defense-related compounds to inhibit the activities of detoxification enzymes in insects. The downregulation of detoxification enzyme activity here suggests the uptake of toxic chemicals was not sufficiently eliminated.

In the present study, PRO activity in GPA upregulated significantly, suggesting that the type or content of nutrients available for GPA was changed in MeJA-treated pepper seedlings. MeJA-mediated plant defense has been reported in many plant species, and the induction of certain anti-nutritional or anti-digestive proteins attributes to the defense response (Chen et al. 2005, Sun et al. 2011). The alteration of the GPA digestive enzymes can improve its digestion and avoid toxicity caused by nutritional imbalance.

Pearson’s correlation analysis could better interpret the relevance between GPA performance and the enzymatic alternation. Different enzymes showed distinct correlation with the development and reproduction indexes of GPA, even in the same enzyme system, and some enzymes were highly positively correlated while others showed low correlation. This phenomenon indicated that certain enzymes might play a primary role in GPA adapting to the significant change in a MeJA-treated host plant. Similarly, Pearson’s correlation analysis was also used to demonstrate the relationship between insect performance and insect enzyme activity, when the plants were treated with JAs. Examples can be seen in the interaction between cotton and cotton bollworm (Yang et al. 2022) and interaction between rose and leaf beetle (Yan et al. 2021), in which specific insect enzymes that significantly attributed to inhibition of insect feeding behavior and growth were clarified. During insect-plant interaction, the activation or inhibition of enzyme in insect was usually correlated to specific chemicals from the plant. Thus, more attention should be paid to specify the potential enzyme-chemical linkage, which will be beneficial for speculating the mechanism of MeJA-driven plant resistance to insect.

### Conclusion

Exogenous application of MeJA significantly induced resistance against GPA in pepper. GPA development and reproduction were significantly suppressed as the aphids fed on MeJA-treated pepper plants. From the insect biochemical perspective, we deduced that the drastic alteration of antioxidant, detoxifying, and digestive enzyme systems might account for the resistance enhancement of pepper. This study suggests MeJA can be used as a potential component for developing an eco-friendly strategy in GPA management.
